# Prevalence of oral mucosal lesions among geriatric dental patients in Egypt: A cross-sectional study

**DOI:** 10.34172/joddd.025.43929

**Published:** 2025-09-30

**Authors:** Dalia Ghalwash, Sara Abd El-Wahed, Ahmed Hamdy

**Affiliations:** ^1^Oral Medicine and Periodontology Department, Faculty of Dentistry, The British University in Egypt, El Sherouk City, Cairo, Egypt; ^2^Oral Pathology Department, Faculty of Dentistry, The British University in Egypt, El Sherouk City, Cairo, Egypt

**Keywords:** Geriatric, Oral health, Oral mucosal lesions, Prevalence, Quality of life

## Abstract

**Background.:**

The oral health needs of older adults must be prioritized due to the shifting demographics of the population and rising life expectancy. This study investigated the prevalence of oral mucosal lesions (OMLs) among geriatric dental patients in a sample of the Egyptian population and explored the association with age, gender, smoking habit, as well as the impact on the quality of life.

**Methods.:**

This cross-sectional study screened 300 geriatric dental patients aged 65 years or older, from several dental hospitals and mobile clinics in various regions in Egypt. Demographic data and information regarding denture use, systemic diseases, and smoking habits were recorded. A clinical examination was conducted, and the quality of life was assessed using the Oral Health Impact Profile-5 (OHIP-5) score.

**Results.:**

OMLs were found in 59.3% of the cases studied. The most prevalent OML in the studied population was coated tongue, and the least frequent was oral cancer. The buccal mucosa was the most frequently affected location. OMLs had a profound impact on the quality of life. Male gender, heavy smoking, the presence of medical conditions, and denture use were significant predictors of the presence of OMLs.

**Conclusion.:**

In the present study, the prevalence of oral lesions in geriatric patients was 59.3%. The most prevalent OML in the studied population was coated tongue, and the least frequent was oral cancer. OMLs had a profound impact on the quality of life. The prevalence of OMLs was found to be strongly linked to systemic diseases, heavy smoking, and male gender.

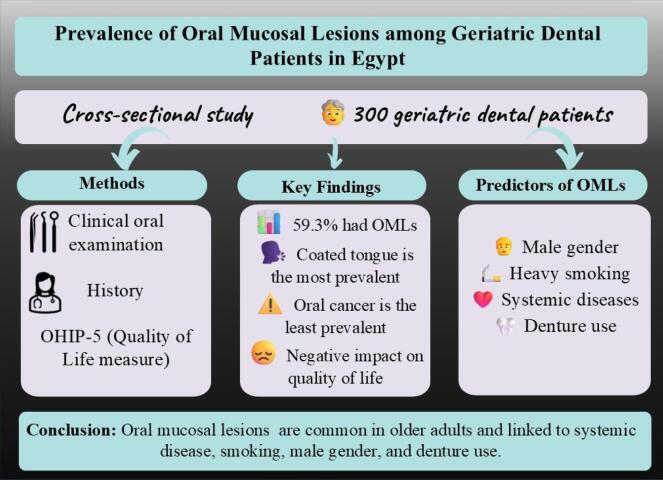

## Introduction

 Significant advances in the medical field have resulted in higher-quality care and improved health outcomes for the global population. As a result, the geriatric population is continuously increasing.^1^ The World Health Organization (WHO) defines older adults as those 65 years or older. According to forecasts, by 2050, one in five people is expected to be over 60 years old.^2^ While this is a substantial success for the medical field, it presents a challenge to many healthcare systems, particularly in developing countries.^1^ Diabetes, hypertension, kidney and liver disease, dementia, and cancer are among the many systemic diseases that affect older adults. Moreover, the physical ability to perform daily activities generally declines with age.^3^

 The oral mucosa in the elderly is subjected to complex environmental factors, leading to age-related changes that modify the pattern of disease presentation in the oral cavity, such as a decrease in cellular density, a reduction in collagen synthesis, atrophy of oral epithelium, and impaired tissue regeneration.^4^ These factors will aggravate damage to the oral mucosal epithelium in response to any irritants. Additionally, this decline in the protective functions of the oral mucosa increases susceptibility to pathogens and exposure to noxious substances, creating an environment prone to the development of various lesions. Therefore, the oral cavity of older adults differs from that of younger individuals.^5,6^ The most common oral conditions observed in the geriatric population include periodontal diseases,^7,8^ decreased salivary flow, chronic oro-facial pain, oral mucosal lesions (OMLs), as well as the presence of precancerous and cancerous lesions.^9,10^ The impact of these lesions not only extends to oral functions, like speech and eating, but also extends to overall health and quality of life.^11,12^

 The presence of comorbid conditions and age-related metabolic changes in elderly individuals makes them more susceptible to oral health issues.^3,5^ The prevalence of systemic diseases, nutritional deficiencies, and deleterious habits further compounds the risk of developing OMLs. Both normal aging changes and disease-related factors can contribute to the development of oral lesions.^13,14^

 The OMLs seen in elderly patients vary by country, region within a country, and even among different communities. Therefore, understanding the prevalence of OMLs in various populations is essential for evaluating treatment needs and providing personalized patient care.^14^ In Egypt, the elderly population was nearly 3.96 million in March 2022^15^ and was estimated to be 4.03 million in January 2023,^16^ indicating an increasing trend in this age group in Egypt. However, there is a significant gap in current research regarding the oral health status of older people, which warrants attention.

 Recognizing the importance of addressing oral health in the elderly population, and the shortage of epidemiological research concerning the prevalence of OMLs in the geriatric people in Egypt, this study aimed to investigate the prevalence OMLs among geriatric dental patients in a sample of the Egyptian population and to explore the association between the prevalence and distribution of OMLs with age, gender, smoking habit, as well as the impact on the quality of life.

## Methods

###  Sample size

 Based on a recent article investigating the prevalence and distribution of OMLs among geriatric patients in India,^5^ the prevalence of oral OMLs in geriatric patients was 40% out of 600 patients assessed. By setting alpha at 0.05 and beta at 0.2, the minimum sample size to achieve statistical power was 229. To accommodate drop-out and attrition, the sample size was increased by 20%, resulting in 270 patients.

 This cross-sectional study screened patients from several dental hospitals and mobile clinics in various regions in Egypt from January to June 2025. Consecutive sampling was used to reduce selection bias. The study enrolled 300 geriatric dental patients aged ≥ 65, including both male and female patients from Egypt. Exclusion criteria included patients younger than 65 years, uncooperative or unwilling older adults, and non-Egyptian individuals. After a detailed explanation of the research scope, patients who agreed to participate in the study signed a written informed consent form before the interview and examination. The study was conducted in accordance with the World Medical Association’s Code of Ethics (Declaration of Helsinki) for experiments involving humans and was approved by the Ethics Committee of the Faculty of Dentistry, with approval number 25-025. The reporting of this study conforms to STROBE guidelines.

 Along with the patients’ demographic details, information regarding denture use, systemic diseases, and smoking habits was recorded. Smokers were divided into light smokers (smoking 1–10 cigarettes/day), moderate smokers (smoking 11–20 cigarettes/day), and heavy smokers (smoking more than 20 cigarettes/day).

 Clinical examination was conducted based on visual inspection and palpation for the whole oral cavity to detect any OMLs by only one well-trained dental professional. To improve daylight and standardize lighting conditions, a visual inspection was conducted in the morning. Clinical oral examination followed the biosafety standards of the WHO using a sterilized probe and mirror, mask, gloves, and gauze pads. The diagnosis of OMLs was made by analyzing the patient’s history and clinical examination findings, and the type and location of the OMLs were recorded. Regarding leukoplakia and oral lichen planus (OLP), diagnoses were made according to a consensus report from the WHO Collaborating Centre for Oral Cancer, and histopathological confirmation was performed when necessary.^17^

 Oral health-related quality of life was measured using a validated Arabic version of Oral Health Impact Profile-5 (OHIP-5).^18^ OHIP-5 scores range from 0 to 20, with higher scores indicating a worse impact on the quality of life.^19^

###  Statistical analysis and data interpretation

 Data analyses were performed using SPSS 26 (SPSS Inc., PASW Statistics for Windows version 26. Chicago: SPSS Inc.). Categorical variables were described using frequencies and percentages, and continuous variables were described using mean ± standard deviation (SD) or median (IQR), as appropriate. The level of statistical significance for the results was set at *P* < 0.05. Normality of continuous variables was assessed using the Kolmogorov–Smirnov test.

- The chi-square test was used to assess associations between categorical variables.
- The Mann-Whitney U test was used to compare 2 independent groups for non-normally distributed continuous variables.
- The Kruskal-Wallis test was used to compare more than 2 independent groups for non-normally distributed continuous data.
- Binary logistic regression was conducted to identify independent predictors of OMLs, and adjusted odds ratios (AORs) with 95% confidence intervals (CIs) were reported.

## Results

###  Participant characteristics

 The present study included 300 geriatric dental patients, with 164 males and 136 females. Patients were categorized into four age groups: 65‒70 years, 71‒75 years, 76‒80 years, and > 80 years. Table 1 presents the demographic characteristics and medical histories of the studied sample.

**Table 1:** Demographic characteristics of the studied sample

	**Groups**	**N=300**	**%**
Age (y)	65‒70	159	53.0
	71‒75	58	19.3
	67‒80	57	19.0
	> 80	26	8.7
Sex	Male	164	54.7
	Female	136	45.3
Smoking	-VE	201	67.0
	Moderate	23	7.7
	Heavy	76	25.3
Medical history	Hypertension	107	35.7
	Diabetes	81	27.0
	Renal disease	26	8.7
	Liver disease	21	7.0
	CVD	18	6.0
Denture use	-	28	9.3

###  Prevalence of OMLs

**Figure 1:**
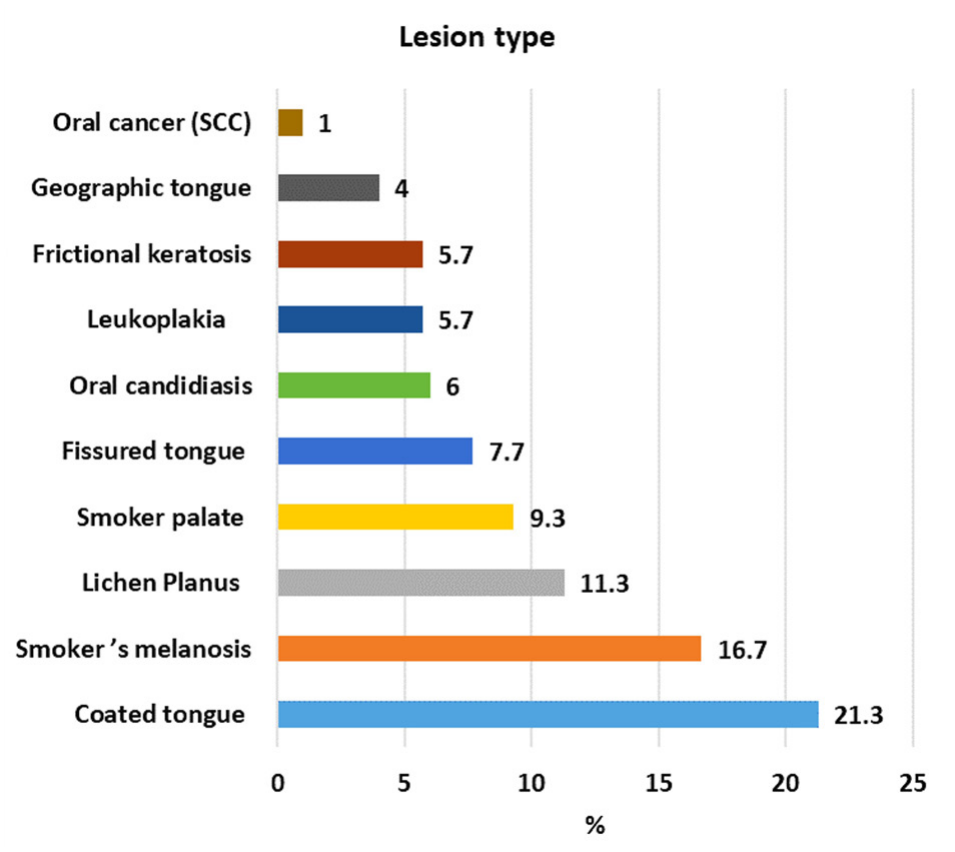
Prevalence of all the types of OMLs

 OMLs were found in 59.3% of the cases studied (178/300) (Figure 1). The 178 patients with OMLs included 114 males (64%) and 64 females (36%); 91 OMLs were found in the age group 65–70 years, with 35 in the 71‒75, 36 in the 76‒80, and 16 in the > 80 age groups.

###  Association with risk factors

 Smoking was reported by 99 patients, and 97 of them had OMLs. Out of these, 23 were moderate smokers and 74 were heavy smokers. Systemic diseases were reported by 218 of the total geriatric population and 156 out of 178 who presented with OMLs (87.6%), and the commonest diseases were hypertension and diabetes, followed by renal, liver, and cardiovascular diseases. Denture use was reported by 28 patients, 25 of whom had OMLs. A significant association was found between OMLs and diabetes, hypertension, denture use, and higher OHIP-5 scores (Table 2).

**Table 2:** Relation between the presence of oral mucosal lesions and demographic data, medical history, and quality of life score

	**Groups**	**Total number**	**Oral mucosal lesions**		**Test of significance**	***P*** ** value**
			**No lesion**	**Lesion**		
Age (y)	65‒70	159	68 (42.8)	91 (57.2)	χ^2^ = 0.713	0.870
	71‒75	58	23 (39.7)	35 (60.3)		
	67‒80	57	21 (36.8)	36 (63.2)		
	> 80	26	10 (38.5)	16 (61.5)		
Sex	Male	164	50 (30.5)	114 (69.5)	χ^2^ = 15.53	0.001*
	Female	136	72 (52.9)	64 (47.1)		
Smoking	-VE	201	120 (59.7)	81 (40.3)	χ^2^ = 91.51	0.001*
	Moderate	23	0	23 (100)		
	Heavy	76	2 (2.6)	74 (97.4)		
Medical history	-VE	82	60 (73.2)	22 (26.8)	χ^2^ = 49.41	0.001*
	+ VE	218	62 (28.4)	156 (71.6)		
Medical history	Hypertension	107	34 (31.8)	73 (68.2)	χ^2^ = 5.45	0.02*
	Diabetes	81	8 (9.9)	73 (90.1)	χ^2^ = 43.59	0.001*
	Renal disease	26	11 (42.3)	15 (57.7)	χ^2^ = 0.032	0.859
	Liver disease	21	11 (52.4)	10 (47.6)	χ^2^ = 1.28	0.257
	CVD	18	10 (55.6)	8 (44.4)	χ^2^ = 1.86	0.185
Denture use	-	28	3 (10.7)	25 (89.3)	χ^2^ = 11.48	0.001*
OHIP-5	Mean ± SD	3.19 ± 2.34	1.43 ± 0.91	4.39 ± 2.27	KW = 13.65	0.001*

OHIP-5: Oral Health Impact Profile-5. χ^2^: chi-squared test, KW: Kruskal-Wallis test,*Statistically significant.

###  Lesion distribution

**Figure 2:**
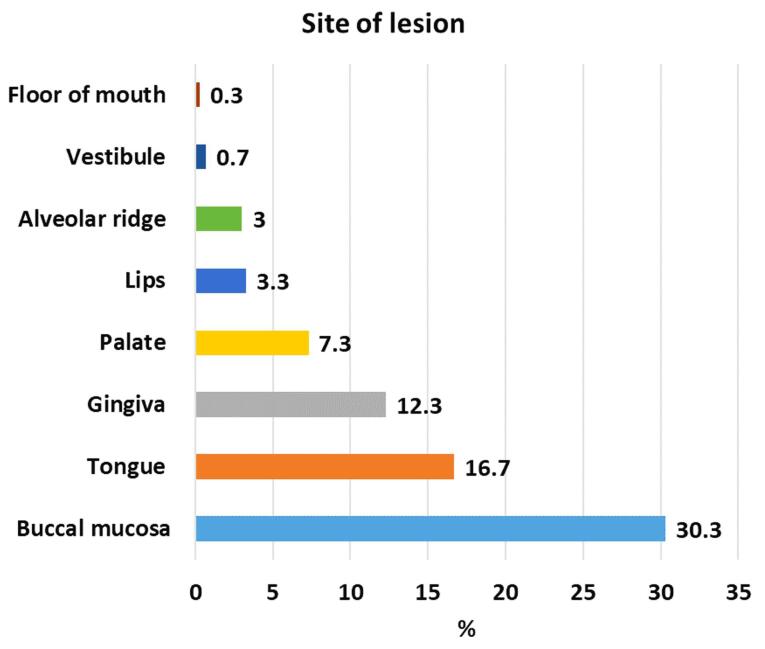
Distribution of OMLs according to the site affected.

 In the present study, the most frequently encountered OMLs were coated tongue (21.3%), followed by smoker’s melanosis (16.7%), OLP (11.3%), smoker’s palate (9.3), fissured tongue (7.7%), oral candidiasis (6%), leukoplakia (5.7%), frictional keratosis (5%), geographic tongue (4%), and oral cancer (1%). The most involved locations were the buccal mucosa, followed by the tongue and the gingiva (Figures 1 and 2).

 A significant association was also encountered between the presence of multiple OMLs and male gender (*P* = 0.001), heavy smoking (*P* = 0.001), and the presence of medical conditions (*P* = 0.001) (Table 3).

**Table 3:** Relation between the number of oral mucosal lesions and demographic data, medical history, and quality of life score

	**Groups**	**Total number**	**Oral mucosal lesions**			**Test of significance**	***P*** ** value**
			**No lesion**	**Single**	**Multiple**		
Age (**y**)	65-70	159	68 (42.8)	56 (35.2)	35 (22)	χ^2^ = 2.32	0.887
	71-75	58	23 (39.7)	21 (36.2)	14 (24.1)		
	67-80	57	21 (36.8)	18 (31.6)	18 (31.6)		
	> 80	26	10 (38.5)	10 (38.5)	6 (23.1)		
Sex	Male	164	50 (30.5)	56 (34.1)	58 (35.4)	χ^2^ = 27.38	0.001*
	Female	136	72 (52.9)	49 (36)	15 (11)		
Smoking	-VE	201	120 (59.7)	72 (35.8)	9 (4.5)	χ^2^ = 153.12	0.001*
	Moderate	23	0	8 (34.8)	15 (65.2)		
	Heavy	76	2 (2.6)	25 (32.9)	49 (64.5)		
Medical history	-VE	82	60 (73.2)	20 (24.4)	2 (2.4)	χ^2^ = 55.18	0.001*
	+ VE	218	62 (28.4)	85 (39)	71 (32.6)		
Medical history	Hypertension	107	34 (31.8)	35 (32.7)	38 (35.5)	χ^2^ = 12.02	0.002*
	Diabetes	81	8 (9.9)	42 (51.9)	31 (38.3)	χ^2^ = 43.72	0.001*
	Renal disease	26	11 (42.3)	8 (30.8)	7 (26.9)	χ^2^ = 0.243	0.886
	Liver disease	21	11 (52.4)	8 (38.1)	2 (9.5)	χ^2^ = 2.85	0.239
	CVD	18	10 (55.6)	8 (44.4)	0	χ^2^ = 6.19	0.045*
Denture use		28	3 (10.7)	17(60.7)	8(28.6)	χ^2^ = 12.88	0.002*
OHIP-5	Mean ± SD	3.19 ± 2.34	1.43 ± 0.91	4.48 ± 2.47	4.26 ± 1.94	KW = 64.5	0.001*

OHIP-5: Oral Health Impact Profile-5. χ^2^: chi-squared test, KW: Kruskal-Wallis test,*Statistically significant.

 Male gender was significantly associated with coated tongue, smoker’s melanosis, smoker’s palate, and leukoplakia. The female gender was significantly associated with oral candidiasis and OLP (Table 4). When the location of OMLs was considered, the palate (*P* = 0.008), alveolar ridge (*P* = 0.006), and gingiva (*P* = 0.042) were significantly associated with male gender, while the tongue was significantly associated with female gender (*P* = 0.049).

**Table 4:** Relation between the type of oral mucosal lesions and the sex of the studied cases

	**Total number**	**Sex**		**Test of significance**	***P*** ** value**
		**Male**	**Female**		
Geographic tongue	12	8 (66.7)	4 (33.3)	χ^2^ = 0.426	0.394
Coated tongue	64	49 (76.6)	15 (23.4)	χ^2^ = 15.74	0.001*
Fissured tongue	23	14 (60.9)	9 (39.1)	χ^2^ = 0.387	0.534
Smoker palate	28	24 (85.7)	4 (14.3)	χ^2^ = 12.02	0.001*
Smoker’s melanosis	50	43 (86)	7 (14)	χ^2^ = 23.77	0.001*
Oral candidiasis	18	3 (16.7)	15 (83.3)	χ^2^ = 11.15	0.001*
Lichen Planus	34	11 (32.4)	23 (67.6)	χ^2^ = 7.70	0.006*
Leukoplakia	17	16 (94.1)	1 (5.9)	χ^2^ = 11.32	0.001*
Frictional keratosis	15	9 (60)	6 (40)	χ^2^ = 0.181	0.670
Oral cancer (SCC)	3	2 (66.7)	1 (33.3)	χ^2^ = 0.176	1.0

χ^2^: chi-squared test,*Statistically significant.

 Heavy smoking was significantly associated with the presence of smoker’s melanosis, smoker’s palate, fissured tongue, leukoplakia, and geographic tongue (Table 5). It was also significantly associated with the location of OMLs on the buccal mucosa (P = 0.001), palate (*P* = 0.001), alveolar ridge (*P* = 0.001), lips (*P* = 0.019), and gingiva (*P* = 0.001).

**Table 5:** Relation between smoking history and the type of oral mucosal lesions

	**Total number**	**Smoking**			**Test of significance**	***P*** ** value**
		**No**	**Moderate**	**Heavy**		
Geographic tongue	12	0	4 (33.3)	8 (66.7)	χ^2^ = 27.55	0.001*
Coated tongue	64	28 (43.8)	12 (18.8)	24 (37.5)	χ^2^ = 24.35	0.001*
Fissured tongue	23	10 (43.5)	4 (17.4)	9 (39.1)	χ^2^ = 7.0	0.03*
Smoker palate	28	0	5 (17.9)	23 (82.1)	χ^2^ = 64.22	0.001*
Smoker’s melanosis	50	0	11 (22)	39 (78)	χ^2^ = 121.97	0.001*
Oral candidiasis	18	17 (94.4)	0	1 (5.6)	χ^2^ = 6.58	0.037*
Lichen Planus	34	25 (73.5)	0	9 (26.5)	χ^2^ = 3.20	0.202
Leukoplakia	17	2 (11.8)	3 (17.6)	12 (70.6)	χ^2^ = 25.12	0.001*
Frictional keratosis	15	6 (40)	3 (20)	6 (40)	χ^2^ = 6.19	0.045*
Oral cancer (SCC)	3	2 (66.7)	0	1 (33.3)	χ^2^ = 0.309	0.857

χ^2^: chi-squared test,*Statistically significant.

Table 6 demonstrates that the statistically significant predictors of the presence of OMLs in the studied cases were male gender, heavy smoking, the presence of medical conditions, and denture use.

**Table 6:** Predictors of the presence of OMLs among the studied cases

	**Groups**	**β**	***P*** ** value**	**AOR (95% CI)**
Age (years)	65‒70		0.870	R
	71‒75	0.129	0.681	1.14 (0.616‒2.09)
	67‒80	0.248	0.436	1.28 (0.687‒2.38)
	> 80	0.179	0.680	1.19 (0.511‒2.79)
Sex	Male		0.942	2.56 (1.59‒4.12)
	Female	< 0.001*		R
Smoking	-VE		0.001*	R
	Moderate	21.59	0.998	UNDEFINED
	Heavy	4.0	0.001*	54.82 (13.08‒229.63)
Medical history	-VE		0.001*	R
	+ VE	1.92		6.86 (3.88‒12.14)
Denture use	-	1.86	0.003*	6.48 (1.91‒21.98)

Model fit Log likelihood = 275.378 Cox & Snell R Square = 0.352 Nagelkerke R Square = 0.475 Omnibus Tests of Model Coefficients = 129.99, *P* < 0.001* Hosmer and Lemeshow Test = 3.89, P = 0.792. β: regression coefficient, AOR (adjusted odds ratio).

###  OHIP-5 analysis

Table 7 shows that the OHIP-5 scores were significantly higher among participants with multiple OMLs, indicating a worse quality of life. Specifically, lesions such as oral candidiasis, leukoplakia, OLP, and oral cancer were strongly associated with elevated OHIP-5 scores. Anatomical sites, including the buccal mucosa, vestibule, tongue, and gingiva, were also significantly linked to a greater negative impact on oral health-related quality of life.

**Table 7:** Relation between the OHIP score and demographic data, medical history, and the number, type, and site of oral mucosal lesions

	**Groups**	**OHIP**	**Test of significance**
Age (y)	65‒70	2.84 ± 2.07	KW = 4.83 *P* = 0.185
	71‒75	3.52 ± 2.55	
	67‒80	3.81 ± 2.74	
	> 80	3.23 ± 2.23	
Sex	Male	3.16 ± 2.12	Z = 0.179 *P* = 0.858
	Female	3.21 ± 2.58	
Smoking	-VE	2.81 ± 2.43	KW = 9.76 *P* = 0.002*
	Moderate	3.43 ± 1.38	
	Heavy	4.12 ± 2.06	
Medical history	-VE	2.06 ± 1.70	Z = 5.33 *P* = 0.001*
	+ VE	3.61 ± 2.41	
Oral mucosal lesions	No lesion	1.43 ± 0.91	Z = 13.65 *P* = 0.001*
	Lesion	4.39 ± 2.27	
Oral mucosal lesions	No lesion	1.43 ± 0.91	KW = 169.21 *P* = 0.001*
	single	4.48 ± 2.47	
	multiple	4.26 ± 1.94	
Oral mucosal lesions	Geographic tongue	3.75 ± 0.62	Z = 0.647, *P* = 0.394
	Coated tongue	3.13 ± 1.80	Z = 0.245, *P* = 0.813
	Fissured tongue	3.13 ± 2.39	Z = 1.92, *P* = 0.142
	Smoker palate	3.89 ± 1.70	Z = 1.94,* P* = 0.09
	Smoker’s melanosis	3.12 ± 2.44	Z = 0.858, *P* = 0.301
	Oral candidiasis	5.06 ± 1.83	Z = 5.5, *P* = 0.001*
	Lichen Planus	7.62 ± 1.82	Z = 6.5, *P* = 0.001*
	Leukoplakia	6.12 ± 0.85	Z = 2.9, *P* = 0.001*
	Oral cancer (SCC)	8.33 ± 1.16	Z = 4.5, *P* = 0.001*
	Frictional keratosis	3.80 ± 1.26	Z = 0.968*, P* = 0.298
Site of lesion	Buccal mucosa	5.44 ± 2.28	Z = 5.8, *P* = 0.001*
	Vestibule	6.50 ± 0.71	Z = 1.99, *P* = 0.045*
	Floor of the mouth	6.0 ± 0.0	Z = 0.258, *P* = 0.230
	Tongue	6.10 ± 2.19	Z = 6.2, *P* < 0.001*
	Palate	3.68 ± 0.945	Z = 0.365,* P* = 0.304
	Alveolar ridge	5.33 ± 1.32	Z = 4.39, *P* = 0.005*
	Gingiva	5.24 ± 2.38	Z = 7.9, *P* = 0.001*
	Lips	4.50 ± 2.51	Z = 1.88, *P* = 0.071

KW: Kruskal-Wallis test, *Statistically significant, Z: Mann-Whitney U test.

## Discussion

 OMLs are quite common in the general population. This prevalence is even higher among the elderly,^5,11,20^ due to aging, metabolic changes, systemic health issues, nutritional deficiencies, use of prosthetics, medications, and smoking. Therefore, the oral health of this population should be a primary focus in effective oral health care services.

 Studies have shown a wide variation in the prevalence of OMLs in geriatric patients, ranging from 7.19%^21^ to 87.6%.^22^ The variation is primarily due to differences in methodologies, sampling methods, and demographic characteristics in different populations. In the present study, OMLs were found in 59.3% of the cases studied, which is similar to several studies conducted in Venezuela (57.0%),^23^ India 54.66%,^24^ Spain (51.1%),^25^ and Iran (52.5%).^20^ This prevalence is high compared to that in studies from Lebanon (22.8%),^26^ Turkey (15.5%),^27^ Saudi Arabia (15.0%),^28^ and Thailand (7.19%).^21^ It is lower than reports from Turkey (87.6%)^22^ and Iran (86.1%).^29^

 The most common OMLs in older adults vary across studies. In this study, the most frequently observed OML was coated tongue (21.3%). Similar to our findings, the most common oral condition in Iran, Indonesia, India, and Spain was coated tongue.^14,20,30,31^ The high incidence of coated tongue may be attributed to poor oral hygiene maintenance in elderly individuals or as a side effect of certain medications.^20^ A coated tongue can serve as an ideal environment for producing malodorous compounds, thereby predisposing individuals to halitosis. Therefore, coated tongue should be appropriately managed in geriatric patients to improve oral hygiene, reduce discomfort, and enhance self-confidence.^31^ The buccal mucosa (30.3%) was the most affected area, which is consistent with the findings of several studies.^5,14,32,33^

 OML cases were closely linked to male gender, with 64% occurring among males and 36% among females, aligning with previous research reporting a male predilection of 56.2%,^34^ 66%,^35^ and 68%.^5^ This may be explained by greater exposure of males to risky habits that affect their oral health, compared to females, according to the social values of our community. Conversely, some studies have reported a higher prevalence of OMLs in females.^23,36^

 Male gender was also significantly linked to the presence of multiple OMLs, particularly coated tongue, smoker’s melanosis, smoker’s palate, and leukoplakia, aligning with the male predilection for smoking in Egypt.^37^ In the present study, smoking was also significantly associated with the presence of multiple OMLs, consistent with several studies.^20,38^ In contrast, one study failed to find a strong relationship between OMLs and smoking.^21^ It was also significantly associated with the location of OMLs on the buccal mucosa, palate, alveolar ridge, lips, and gingiva. Smoker’s melanosis and smoker’s palate were exclusively encountered in smokers in the present study, reflecting the strong correlation with the habit in older adults.

 Systemic diseases were reported in 87.6% of patients with OMLs; they were strongly linked to the presence of multiple OMLs, aligning with previous research.^22^ OMLs were notably associated with hypertension and diabetes in this study, consistent with several earlier studies.^5,14,20,21^

 A decrease in the incidence of OMLs was observed with increasing age, consistent with previous studies.^21,32,34,39,40^ The highest prevalence of OMLs was identified among those aged 65–70 years (91 OMLs), followed by the 71‒75 (35 OMLs), 76‒80 (36 OMLs), and > 80 (16 OMLs) age groups. This decline in OML incidence with age may result from reduced smoking habits related to age-associated health issues, which lower the risk of OML development. The 65‒70-year group (159/300) had a higher number of patients than 58/300, 57/300, and 26/300 in the three older age groups, which is another possible explanation.

 The oral cavity undergoes gradual, irreversible, and cumulative changes due to aging, which makes it more susceptible to traumatic and infectious agents. Oral candidiasis was identified in 6% of the studied sample, showing a significant association with female gender. The findings are consistent with studies of elderly populations, indicating that females are more likely to have oral candidiasis.^36,39^ This is likely due to changes in salivary function and oral microbiota, as both the quantity and composition of saliva change with age, along with increased medication intake and denture wear.

 In the present study, a significant association was found between denture use and OMLs, particularly oral candidiasis, consistent with several previous studies.^11,14,22,26^ The reason for this is that micropores can develop acrylic resin over time, allowing microorganisms to colonize the prosthesis.^26^ The consistent use of the prosthesis can lead to mechanical irritation and infection.^22,26^ According to previous research, female subjects are more susceptible to denture-related lesions.^38^ This may be due to hormonal changes, which cause atrophy of the oral mucosa and reduce protection against the chronic irritation from poorly fitting dentures.^32,38^

 Given the potential implications of OMLs, which include oral cancer and precancerous lesions, understanding their prevalence and epidemiological traits is crucial for maintaining the overall health of the geriatric population.^41^ OLP is a potentially malignant disorder with a reported rate of epithelial dysplasia of 10.19% according to a recent study in Egypt.^42^ OLP was observed in 11.3% of our patients, compared to 17% in a previous study conducted in India.^35^ Nevertheless, other studies reported a prevalence rate of 3% and 0.8%.^23,27^ Diabetes mellitus and hypertension were strongly associated with OMLs in this study and are strongly linked to OLP according to the literature, along with the medications used for their treatment, which could predispose patients to develop OLP.^5,24^ Female gender showed a significant association with OLP in the present study, aligning with numerous studies that report a female predominance with OLP.^24,39,41^ The connection between OLP in women is often attributed to hormonal changes and psychological stress.^24^

 Leukoplakia was encountered in 5.7% of the cases studied and was strongly linked to male gender, consistent with previous reports of a male predominance in leukoplakia.^24,43^ Leukoplakia was also significantly associated with smoking habit, in line with most literature.^32,39^

 The oral cavity is a prime location for the development of cancerous lesions. Older individuals in many countries have an increased incidence of oral cancer in men than in women, leading to concerns about oral health care among the elderly population. In the present investigation, two males and one female patient, comprising 1% of the studied sample, were diagnosed with oral cancer (SCC), while oral cancer involved 2% and 6.66% of the elderly population in Indian studies.^32,35^ The higher prevalence could be explained by the increased consumption of tobacco and products related to it, even in older age, in India. Early detection of suspicious oral precancerous lesions by screening examination and timely interventions is crucial for maintaining health;^10,41^ however, the elderly population faces delays in diagnosis due to limited access to oral health care and a lack of awareness about the harmful effects of smoking.

 According to the binary logistic regression analysis of our data, the statistically significant predictors of the presence of OMLs among the studied cases were male gender, heavy smoking, the presence of medical conditions, and denture use. Males had a 2.56 times higher risk of developing OMLs than females. Heavy smokers had a 54.82 times higher risk of developing OMLs than nonsmokers, and the presence of a medical condition and history of denture use were also associated with a 6.86- and 6.48-times higher risk of having OMLs. Therefore, personalized care should be given to geriatric patients, and particularly males should be encouraged to quit smoking, as smoking cessation significantly reduces the risk of developing OMLs and oral cancer.^11,40^

 OMLs negatively influence the patients’ quality of life due to increased difficulties with eating, speaking, and daily activities, leading to functional and psychosocial problems.^44^ Thus, it is important to assess the impact of oral health on patients’ quality of life.^5^ In the present study, the OHIP-5 scores were significantly higher among participants with multiple OMLs, indicating a worse quality of life, which aligned with several studies.^5,11,44,45^ Additionally, age, heavy smoking, and medical conditions were also strongly associated with higher OHIP scores. Oral candidiasis, OLP, leukoplakia, and oral cancer were strongly linked to a greater negative impact on oral health-related quality of life in our results. Therefore, early detection of these OMLs is crucial for prognosis and treatment. Clinicians need to recognize, diagnose, and treat OMLs that occur in older adults, as this can significantly enhance clinical outcomes and patients’ quality of life.

 Oral health care in the geriatric population is often neglected, especially in low- and middle-income countries like Egypt, where older patients may only visit the dentist if they have a problem or may never do so at all.^12^ These findings highlight the importance of incorporating more geriatric dentistry training into the dental education curriculum in Egypt to better prepare future dental professionals with the necessary specialized knowledge and skills to provide optimal care for this vulnerable group.

 The current investigation revealed the epidemiological characteristics of OMLs in geriatric dental patients. Since most OMLs were associated with smoking, smokers were advised to quit the habit because of its harmful influence on oral and general health and the risk of oral cancer. Patients with precancerous lesions, such as leukoplakia and OLP, will undergo periodic clinical examinations to detect any potential malignant transformation, which is especially relevant in the elderly population. Moreover, the geriatric population should be educated through community-based programs to get screened for any OMLs. Enhancing geriatric dentistry training in Egypt’s dental education system is essential for preparing future dental professionals to meet the needs of an aging population. This would not only improve clinical outcomes but also significantly enhance the quality of life for this vulnerable group.

## Limitations

 The limited sample size is one of the limitations of the present cross-sectional study. The absence of a detailed medication use history hindered the ability to correlate it with the prevalence or characteristics of opioid misuse disorder. Additionally, the nature of the cross-sectional design limits causal inference; only associations between variables can be observed. Information regarding smoking habits and medical conditions was self-reported by participants and could be subject to recall or social desirability bias. Regarding examiner calibration, only one examiner conducted all the clinical examinations to ensure consistency; however, examiner calibration or inter-examiner reliability measures were not formally performed, which may impact diagnostic reproducibility.

 Another limitation is that random sampling was not feasible or practical due to the nature of the study setting. Participants were recruited from multiple dental hospitals and mobile clinics that serve diverse and often underserved populations across different regions. These clinics operate on either a walk-in or scheduled visit system, making it logistically challenging to create a complete sampling frame for random selection. Additionally, our ability to randomly select from a larger population was impeded by resource and time constraints. To minimize selection bias and ensure all eligible patients were included during the study period, we used consecutive sampling.

 Further investigation is needed to validate the associations between demographic factors, systemic diseases, and the occurrence of specific oral OMLs. Future research should prioritize determining the exact prevalence of each condition by using specific lesion types and distinct age groups.

## Conclusion

 In this cross-sectional study, OMLs were identified in 59.3% of geriatric dental patients, with coated tongue being the most common and oral cancer the least frequent. The presence of OMLs was significantly associated with male gender, heavy smoking, systemic medical conditions, and denture use. These findings also revealed that OMLs had a considerable negative impact on patients’ oral health-related quality of life. However, these conclusions should be interpreted with caution due to the study’s limitations, including its cross-sectional design, reliance on self-reported data for smoking and medical history, and the absence of examiner calibration. Further longitudinal and clinical studies with diagnostic validation are advised to enhance our understanding of causal relationships and improve care strategies for the elderly population.

## Competing Interests

 The authors declare no competing interests.

## Ethical Approval

 The study was conducted in accordance with the Declaration of Helsinki for experiments involving humans and was approved by the Ethics Committee of the Faculty of Dentistry, with approval number 25-025.
